# Counteracting Akt Activation by HIV Protease Inhibitors in Monocytes/Macrophages

**DOI:** 10.3390/v10040190

**Published:** 2018-04-13

**Authors:** Sébastien Pasquereau, Amit Kumar, Wasim Abbas, Georges Herbein

**Affiliations:** 1 Pathogens & Inflammation/EPILAB Laboratory, UPRES EA4266, University of Franche-Comté, COMUE Bourgogne Franche-Comté University, 25030 Besançon , France; sebastien.pasquereau@univ-fcomte.fr (S.P.); amit.aiims2005@gmail.com (A.K.); wazim_cemb@hotmail.com (W.A.); 2 Department of Virology, CHRU Besançon, 25030 Besançon, France

**Keywords:** Akt, HIV-1, monocyte, resting CD4+ T cells, cART, protease inhibitor, NNRTI, reservoir

## Abstract

Akt signaling plays a central role in many biological processes that are key players in human immunodeficiency virus 1 (HIV-1) pathogenesis. The persistence of latent reservoirs in successfully treated patients, mainly located in macrophages and latently infected resting CD4+ T cells, remains a major obstacle in HIV-1 eradication. We assessed the in vitro effects of an HIV protease inhibitor (PI) and a non-nucleoside reverse transcriptase inhibitor (NNRTI) on HIV-1 Nef-induced Akt activation in macrophages and on HIV-1 reactivation in U1 monocytoid cells. Ex vivo, we investigated the impact of combination antiretroviral therapy (cART) on Akt activation, as measured by flow cytometry, and on the viral reservoir size, quantified by qPCR, in monocytes and autologous resting CD4+ T cells from HIV-infected individuals (Trial registration: NCT02858414). We found that, in myeloid cells, both Akt activation and HIV-1 reactivation were inhibited by PI but not by NNRTI in vitro. Our results indicate that cART decreases Akt activation and reduces the size of the HIV reservoir in both monocytes and resting CD4+ T cells. Our study indicates that Akt activation could play a role in HIV reservoir formation, indicating that drugs which target Akt could be efficient for limiting its size in aviremic chronically infected patients.

## 1. Introduction

The serine/threonine kinase Akt, also known as protein kinase B, plays important roles in the cellular processes involved in HIV-1 pathogenesis. As a key regulator in the phosphoinositide 3-kinase (PI3K) pathway, it impacts cell survival, metabolism, growth, and proliferation [1]. The HIV-1 infection progression is fuelled by immune hyperactivation, which constitutes a hallmark of the disease. In infected individuals, the tumor necrosis factor (TNF) alpha is one of the proinflammatory cytokines that could be critical for the formation of HIV-1 reservoirs [2,3,4,5].

HIV-1 Nef is an accessory viral protein known to be abundantly expressed in the early stages of infection. Several aspects of the viral pathogenesis have been showed to be affected by this multifunctional protein, which plays an important role despite lacking enzymatic activity [6,7]. Besides endogenously produced Nef, extracellular soluble Nef and/or vesicle-associated Nef are found in the serum or extracellular environment of infected individuals and display immunomodulatory effects such as the suppression of immunoglobulin class switching in bystander B cells and neurotoxicity [8,9,10,11]. The PI3K/Akt pathway, a key pathway of cell survival, is known to be manipulated by a plethora of viral pathogens, including among others human cytomegalovirus, Epstein Barr virus, influenza A virus, and HIV-1 [12]. Several studies have also reported the impact of protease inhibitors (PIs) on Akt signaling in several cell types and in clinical trials [13,14,15,16,17,18,19]. We observed previously a critical role for Nef in the hyperactivation of T cells through Akt activation and that blocking Akt activation especially by HIV-1 protease inhibitors (PIs) could limit HIV-1 recovery from latently infected T cells [20,21].

The use of combination anti-retroviral therapy (cART) has allowed for the reduction of the viral load of infected individuals to undetectable levels [22]. However, in the majority of patients that are considered successfully treated by cART, low levels of active viral replication can be detected by the use of new ultrasensitive assays [23]. A major obstacle to HIV-1 eradication is indeed the persistence of replication-competent proviruses [24,25]. These latent reservoirs are established early during acute viral infection and include, among others, macrophages and latently infected resting CD4+ T cells, these latently infected resting CD4+ T cells being the main viral reservoir [26,27,28,29]. Recently, cART intensification was assessed and did not reduce residual HIV-1 viremia in patients on cART, indicating that its potential to eradicate the virus appears to be limited [30]. In addition, immune hyperactivation, especially TNF production, still persists in aviremic patients under cART and could explain the limited effect of cART on the eradication of HIV reservoirs [31,32].

Initiation of cART during primary HIV infection may limit the establishment of viral reservoirs, and very early cART limits the seeding of the HIV reservoir in long-lived central memory CD4+ T cells [26,33,34]. By contrast, the HIV reservoir in monocytes/macrophages has been much less studied and the impact of cART on the monocyte/macrophage HIV-1 reservoir in aviremic chronically infected patients is so far unknown [29,35,36].

We report here a study indicating that Akt activation by Nef in Monocyte-Derived Macrophages (MDMs) can be blocked by the PI lopinavir/ritonavir, but not by the NNRTI nevirapine. We observed the important role of Akt activation in HIV-1 reactivation from latently infected U1 monocytoid cells in vitro, which could be blocked by PI but not by NNRTI. Finally, in vivo cART-blocked Akt activation in monocytes, but also in resting CD4+ T cells, meanwhile limited the size of the viral reservoir in both cell types isolated from aviremic chronically HIV-1 infected patients.

## 2. Materials and Methods

### 2.1. Isolation and Culture of Peripheral Blood Mononuclear Cells (PBMCs), Peripheral Blood Lymphocytes (PBLs), MDMs, and Resting CD4+ T Cells

Human PBMCs, purified PBLs, and MDMs were prepared from the peripheral blood of healthy donors and cultured as described previously [37]. Purified CD25^−^, CD69^−^, and HLA-DR^−^ CD4+ T cells (resting CD4+ T cells) were obtained from the peripheral blood of HIV^+^ patients using a negative selection assay using magnetic beads (LD columns, Miltenyi Biotec, Bergisch Gladbach, Germany) as described previously [38]. PBMCs were isolated from the peripheral blood of HIV^+^ patients by Ficoll gradient centrifugation as described previously [37] and cultured in Roswell Park Memorial Institute (RPMI) medium supplemented with 10% (*v*/*v*) fetal calf serum. PBLs in suspension and adherent monocytes were collected after 2 h as described previously [37].

### 2.2. Recombinant Nef Treatment

MDMs (5 × 10^6^ cells) were treated with recombinant myristoylated Nef protein (rNef) from the SF2 HIV-1 strain (1–100 ng/mL) (cat # PR-382, Jena Bioscience, Jena, Germany). Cell pellets were collected at various times after rNef treatment, washed extensively, and either lysed before Western blot analysis or fixed with BD Cytofix (BD Biosciences, San Jose, CA, USA) for 20 min before flow cytometric analysis. MDMs were treated with the Akt inhibitor VIII (cat # sc-202048A) (0, 25, 50 µM) (Santa Cruz Biotechnology, Santa Cruz, CA, USA) for 2 h before addition of rNef (100 ng/mL) for 30 min.

### 2.3. Western Blot

Cellular extracts from MDMs treated with rNef were used to examine Akt and pAkt expression by Western blotting as described previously [39]. Anti-Akt-mAb (cat # 2967S, Cell Signaling Technologies, St Quentin, France), anti-pAkt (Thr308) mAb (cat # 2965S, Cell Signaling Technology), anti-pAkt (Ser473) mAb (cat # 4060S, Cell Signaling Technology), and anti-β-actin (Sigma-Aldrich, St. Louis, MO, USA) were used. Antiretroviral drugs (lopinavir/ritonavir and nevirapine) were obtained from the hospital pharmacy of the Centre Hospitalier Régional Universitaire (CHRU) Besançon. MDMs were treated for different periods of time with these drugs at indicated concentrations. The level of pAkt/Akt was determined by immunoblotting.

### 2.4. RT-PCR Assay

For the detection of Akt mRNA using an RT-PCR assay, total RNA was extracted from MDMs either left untreated or treated with increasing concentrations of rNef (1–25 ng/mL) for 30 min using an RNeasy mini kit (Qiagen, Hilden, Germany). A total of 2 µg of RNA was reverse transcribed into cDNA with Superscript IV RT (Life Technologies, Carlsbad, CA, USA) using oligo (dT) primers. The 5 µL of reverse transcription reaction product was amplified using primers against Akt (sense, 5′-ATCCCCTCAACAACTTCTCAGT-3′; antisense, 5′-CTTCCGTCCACTCTTCTCTTTC-3′). The beta-globin gene was amplified as an internal control (sense, 5′-TCCCCTCCTACCCCTACTTTCTA-3′; antisense, 5′-TGCCTGGACTAATCTGCAAGAG-3′). The PCR product was electrophoresed on a 2% agarose gel containing SYBR Green I Stain (Lonza, Rockland, ME, USA).

### 2.5. FACS Analysis

PBLs, monocytes, and resting CD4+ T cells were fixed and permeabilized (BD Cytofix/Cytoperm kit) (BD Biosciences, San Jose, CA, USA). Briefly, 10^6^ cells were thoroughly resuspended in 250 µL of BD Cytofix/Cytoperm solution for 20 min at 4 °C. Cells were washed two times in 1X BD Perm/Wash solution. The cells were pelleted by centrifugation. The cells were thoroughly resuspended in 500 µL of 1X BD Perm/Wash containing PE-conjugated anti-pAkt (cat# 558275)/anti-Akt (cat# 560049) antibodies (BD Biosciences) or isotype control antibody (cat# 551436) (BD Pharmingen). The cells were incubated at 4 °C for 30 min in the dark. The cells were washed three times in 1X BD Perm/Wash solution and resuspended in 1X PBS prior to flow cytometric analysis.

### 2.6. MTT Cell Assay

Cell viability was measured using the MTT assay kit (Cayman Chemical, Ann Arbor, MI, USA). Where specified, MDMs and monocytoid U1 cells stimulated or not with TNF were seeded at a density of 0.1 × 10^6^/well in triplicates in 96-well plates in a final volume of 100 µL medium containing lopinavir/ritonavir, nevirapine, and Akt inhibitor VIII at 50 µM for 0–2 h. MDMs were left untreated or treated with rNef (1–100 ng/mL) for 30 min. After 0–2 h, 10 µL MTT reagent was added to each well and the plates were incubated for another 4 h in the cell culture incubator at 37 °C. The plate was centrifuged at 500 g for 10 min. The cell culture media was aspirated, 100 µL of crystal-dissolving solution was added to each well, and the absorbance was measured at 570 nm using Multiskan Ex (Thermo Electron Corporation, Cergy-Pontoise, France).

### 2.7. Reactivation from Latency in U1 Cells

The monocytoid U1 cell line was obtained from the AIDS Research and Reference Reagent Program (National Institute of Allergy and Infectious Disease (NIAID), National Institute of Health (NIH)). Cells were mock-treated or treated with TNF 10 ng/mL in the absence or presence of lopinavir/ritonavir, nevirapine, and Akt inhibitor VIII at 50 µM. At 24 h post treatment, culture supernatants of U1 cells were harvested and HIV-1 production was measured by determining p24 antigen concentration by ELISA (Innogenetics, Ghent, Belgium) [40]. A WST 1 assay (Roche, Basel, Switzerland) was performed to assess the viability of treated cells.

### 2.8. Quantification of HIV-1 Proviral DNA

DNA was extracted from 10^6^ cells monocytes and resting CD4+ T cells using a QIAamp DNA Blood kit (Qiagen, Hilden, Germany) as per the manufacturer’s instructions. HIV-1 proviral DNA was quantified using a Biocentric Generic HIV DNA Cell kit (Biocentric, Bandol, France) on an MX3005p real-time PCR instrument (Stratagene, San Diego, CA, USA) as per the manufacturers’ instructions.

### 2.9. Patients

We enrolled 31 chronically HIV-1-infected individuals at the Besançon University Hospital (Besançon, France). The patients were treated with cART (treatment range: 1.4–27.2 years) and had undetectable plasma HIV-1 RNA levels (<40 copies/mL) for at least 1 year. These 31 patients treated with cART (PI, *n* = 8; NNRTI, *n* = 23) were studied for levels of Akt activation and HIV-1 proviral DNA in monocytes and autologous resting CD4+ T cells. In addition, we enrolled four HIV-1-infected individuals at the Besançon University Hospital (Besançon, France). These patients were naïve from cART treatment and were studied for HIV-1 proviral DNA in monocytes and autologous resting CD4+ T cells.

### 2.10. Statistical Analyses

The figures show the means and standard deviations for independent experiments. Plotting and statistical analysis were performed using Excel. Results from in vitro reactivation studies and HIV proviral DNA using patient cell cultures of monocytes and resting CD4+ T cells are shown as medians and quartiles. Data sets were analyzed using an unpaired nonparametric *t*-test (Mann–Whitney test) or Wilcoxon *t* test. Differences were considered significant at a value of *p* < 0.05.

### 2.11. Ethics Approval and Consent to Participate

All of the patients who were enrolled at the Besançon University Hospital (France) gave their written informed consent to participate in the study according to the Helsinki declaration. The Human Protection Committee East Area II (CPP EST-2) from France was consulted and approved the study (CPP14/455) (Trial registration number: NCT02858414; Name of registry: Exploratory Study of Cellular Reservoirs in Blood From HIV Infected Patients (EURECA); URL of registry: clinicaltrials.gov; Date of registration: 29 July 2016; Date of enrolment of the first participant to the trial: 9 June 2015; “Retrospectively registered”). This study did not rely solely on medical records. The authors did not have any contact with the study subjects and performed tests on patient blood samples that were part of routine care. The blood samples were anonymized before being used by the authors.

## 3. Results

### 3.1. Recombinant Nef Increases Akt Expression and Phosphorylation in MDMs In Vitro

**Figure 1:**
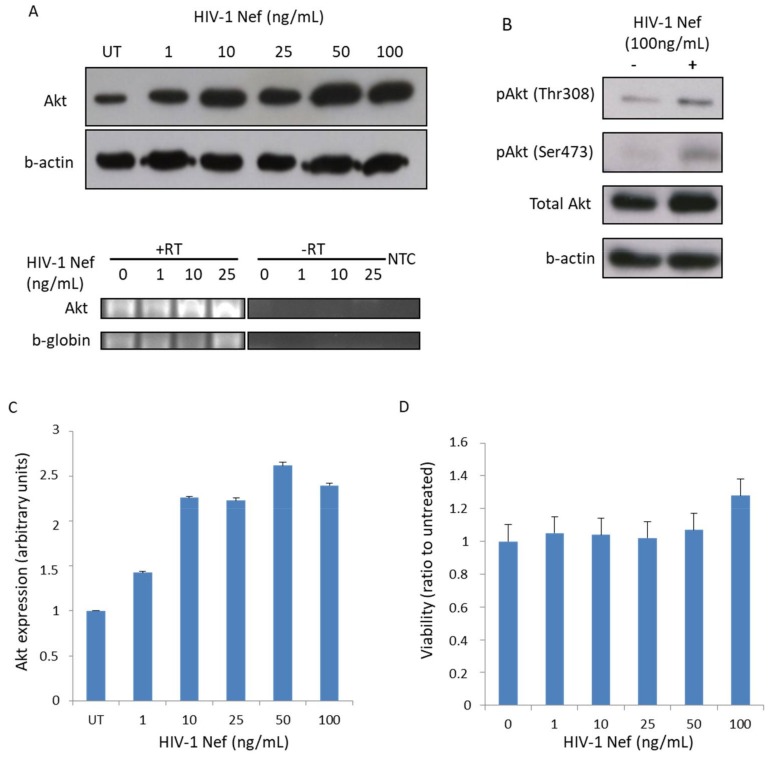
HIV-1 Nef enhances Akt expression and activation in MDMs in vitro. (**A**) Monocyte-Derived Macrophages (MDMs) were either left untreated or treated with increasing concentrations of rNef (1–100 ng/mL) for 30 min. After incubation, protein lysates and RNA extracts were made. *Upper panel.* Expression of total Akt and β-actin was determined by Western blotting. *Lower panel.* Akt mRNA expression was measured by an RT-PCR assay on a 2% agarose gel. Beta-globin was used as an internal control. (**B**) MDMs were either left untreated or treated with rNef (100 ng/mL) for 30 min. After incubation, protein lysates were made and expression of pAkt(Ser473), pAkt(Thr308), and total Akt and β-actin were determined by Western blotting (*n* = 3). (**C**) The histogram shows the enhanced expression of total Akt in MDMs treated with increasing concentrations of Nef for 30 min. UT, untreated. (**D**) No significant toxicity of rNef (1–100 ng/mL) was observed in MDM treated for 30 min as measured by a cell viability assay.

**Figure 2:**
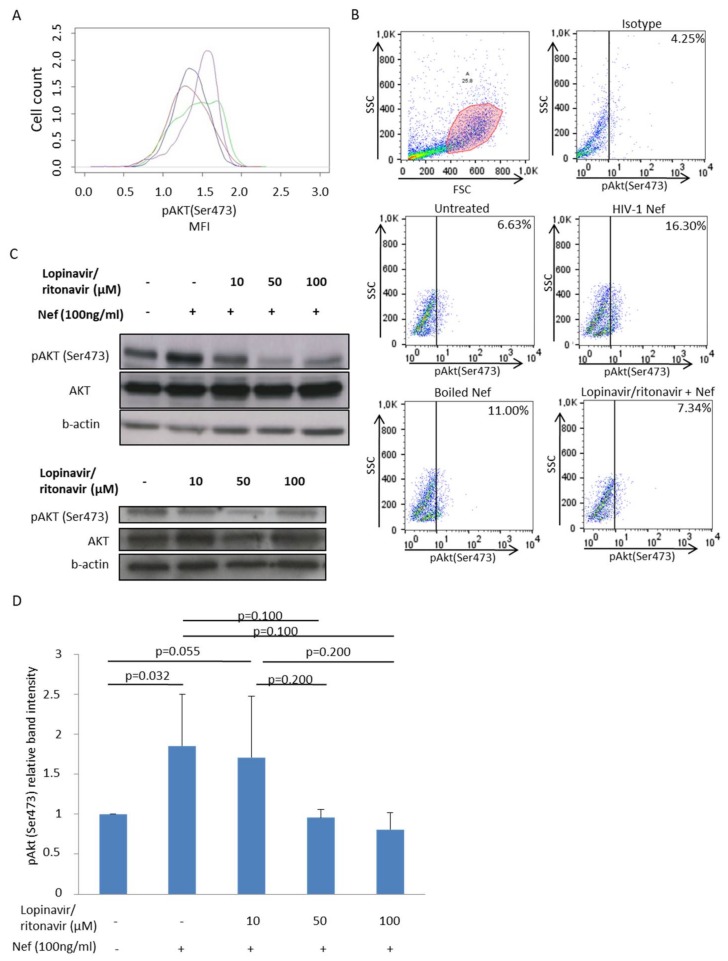
The protease inhibitor (PI) lopinavir/ritonavir blocks Akt activation in MDMs treated with Nef. (**A**) Activation of Akt (pAkt) in MDMs treated with rNef (100 ng/mL) for 30 min is blocked by the PI lopinavir/ritonavir (50 µM) but not by the non-nucleoside reverse transcriptase inhibitor (NNRTI) nevirapine (50 µM) as measured by flow cytometric analysis. Red: untreated MDM, green: MDM treated with Nef, blue: MDM treated with Nef + lopinavir/ritonavir; purple: MDM treated with Nef + nevirapine. Results are representative of three independent experiments. (**B**) The PI lopinavir/ritonavir inhibits Akt activation (pAktSer473) in MDMs treated with rNef as determined by flow cytometric analysis. One million MDMs were treated with rNef (100 ng/mL) for 30 min in the absence or presence of lopinavir/ritonavir (50 µM). Boiled Nef was used as a control. Results are representative of three independent experiments. (**C**) Measurement of Akt activation in MDMs stimulated with rNef (100 ng/mL) or not and treated with increasing concentrations of the PI lopinavir/ritonavir (0–100 µM) as measured by Western blotting. *Upper panel.* Decreased Akt activation in MDMs stimulated with rNef (100 ng/mL) and treated with increasing concentrations of the PI lopinavir/ritonavir (0–100 µM). Beta-actin was used as an internal control. *Lower panel.* Akt activation is slightly inhibited by lopinavir/ritonavir in the absence of Nef treatment. Beta-actin was used as an internal control. Results are representative of three independent experiments. (**D**) Decreased Akt activation in Nef-stimulated MDMs treated with lopinavir/ritonavir is dose-dependent. Histogram represents means (±s.d.) of three independent experiments.

We studied the impact of Nef on both Akt expression and activation in MDMs. We observed that treatment of MDMs with rNef led to enhanced Akt expression in a dose-dependent manner (Figure 1A, upper panel). We performed an RT-PCR assay in order to evaluate mRNA Akt expression in rNef-treated MDMs. We observed enhanced mRNA Akt levels in rNef-treated MDMs compared to untreated MDMs, indicating that the increase in Akt expression after Nef treatment is transcriptional (Figure 1A, lower panel). Akt is activated by its phosphorylation on Ser473 and Thr308 residues [41]. We found that Akt became phosphorylated on serine^473^ and threonine^308^ in MDM treated with rNef as determined by Western blotting and flow cytometry (Figure 1B and Figure 2A). The increased expression of total Akt measured in rNef-treated MDM was dose-dependent (Figure 1C). We did not find any significant toxicity of rNef (1–100 ng/mL) for as long as 30 min as determined by a cell viability assay (Figure 1D).

### 3.2. The Protease Inhibitor (PI) Lopinavir/Ritonavir Blocks Akt Activation in MDMs Treated with rNef in Vitro

Since HIV-1 protease inhibitors (PI), but not reverse transcriptase inhibitors (RTI), have been reported to block Akt activation in several cell types, such as peripheral blood mononuclear cells (PBMCs) and monocytes/macrophages [13,15,16,17,18,19], we assessed the potential role of PIs as immunomodulators in addition to their antiviral effect in HIV-1 infection. In vitro, we observed that the PI lopinavir/ritonavir, but not the non-nucleoside reverse transcriptase inhibitor (NNRTI) nevirapine, inhibited Akt hyperactivation in MDMs stimulated with rNef using flow cytometric analysis (Figure 2A,B). The inhibition of Akt activation by lopinavir/ritonavir was dose-dependent (Figure 2C upper panel and Figure 2D). We observed a slight inhibition of Akt activation by lopinavir/ritonavir in the absence of Nef treatment (Figure 2C lower panel). At the concentrations of anti-HIV drugs used, more than 80–90% of treated cells were viable after two hours of treatment.

### 3.3. The Protease Inhibitor Lopinavir/Ritonavir Limits HIV-1 Reactivation from Chronically Infected U1 Monocytoid Cells Stimulated with TNF via Blocking the Akt Pathway

**Figure 3:**
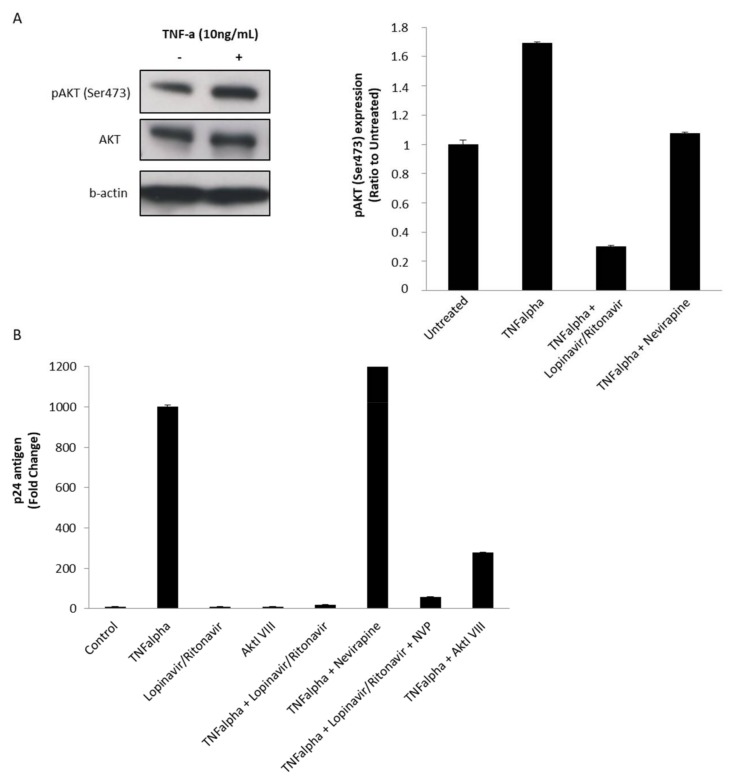
Enhanced Akt activation in monocytoid U1 cells stimulated with Tumor Necrosis Factor (TNF) alpha and blockade of HIV-1 recovery from U1 cells stimulated with TNF and treated with the PI lopinavir/ritonavir. (**A**) *Left panel.* Enhanced Akt activation (pAkt Ser473) in U1 cells treated with TNF (10 ng/mL) for 30 min as measured by Western blot. Beta-actin was used as an internal control. *Right panel.* The histogram shows that increased pAkt levels observed in TNF-stimulated U1 cells are blocked mostly with lopinavir/ritonavir treatment using Western-blot quantification by ImageJ software. Results are representative of three independent experiments. (**B**) U1 cells were either left untreated or treated with TNF(10 ng/mL) in the absence or presence of the PI lopinavir/ritonavir, the NNRTI nevirapine, and the Akt inhibitor VIII (all at 50 µM). The viral production was determined by measuring the level of p24 antigen at 24 h post treatment using ELISA. Means (±s.d.) of three independent experiments are shown.

Since Akt activation participates in immune activation and favours resistance to apoptosis, controlling the Akt pathway using PIs could have an impact on HIV-1 recovery from latently infected monocytoid cells [42,43]. Therefore, we assessed the role of PI treatment on HIV-1 reactivation from latency by measuring viral production in latently infected monocytoid cells U1 treated with TNF. U1 monocytoid cells contain two copies of integrated HIV-1 provirus [44]. In U1 cells stimulated with TNF (10 ng/mL), we observed increased Akt phosphorylation on Ser473 using Western blotting (Figure 3A, left panel). The TNF-induced Akt activation in U1 cells was blocked mostly by treatment with lopinavir/ritonavir (Figure 3A, right panel). In addition, viral reactivation induced by TNF in U1 cells was significantly blocked by treatment with the PI lopinavir/ritonavir and the Akt inhibitor VIII, but not by the NNRTI nevirapine, as measured by p24 detection using ELISA (Figure 3B). Our results indicate that a PI-mediated blockade of Akt activation could limit HIV-1 reactivation of integrated provirus from U1 monocytoid cells especially when driven by TNF.

### 3.4. Decreased Akt Activation in Monocytes from Aviremic Patients under PI-Cart

To further demonstrate the critical role of Akt in HIV-1 pathogenesis and the potential interest of Akt blockade by PI in vivo, we measured Akt activation levels in monocytes and autologous resting CD4+ T cells isolated from HIV-1-positive patients. Thirty-one patients with chronic HIV-1 infection treated with cART (treatment range: 1.4–27.2 years) and with undetectable plasma HIV-1 RNA levels (<40 copies/mL) for at least 1 year were included in the study between 2015 and 2017. Of these thirty-one patients (mean age 47.6 years; range 28–69 years) treated with cART, twenty-three were treated with NNRTI-based cART and eight with PI-based cART (as their most recent treatment) and for more than one year (Table 1). We did not observe significant differences for nadir median CD4 counts (255 × 10^6^ versus 288 × 10^6^ cells/L, *p* = 0.734), median CD4 counts at the initiation of treatment (361 × 10^6^ versus 332 × 10^6^ cells/L, *p* = 0.407), median CD4 counts at the last point (614 × 10^6^ versus 761 × 10^6^ cells/L, *p* = 0·288), and median HIV RNA load at zenith (5.10 versus 4.98 log/mL, *p* = 0.982) between PI-treated and NNRTI-treated patients (Table 1). We did not find significant differences between PI-treated and NNRTI-treated patients for the number of failures of treatment (1.5 versus 1, *p* = 0.694) and the duration of therapy (11.6 versus 9 years, *p* = 0.498). The duration with undetectable plasma HIV-1 RNA levels was not significantly different in the PI-treated arm compared to the NNRTI-treated arm (5.8 versus 6.5 years, *p* = 0.804) (Table 1).

**Figure 4:**
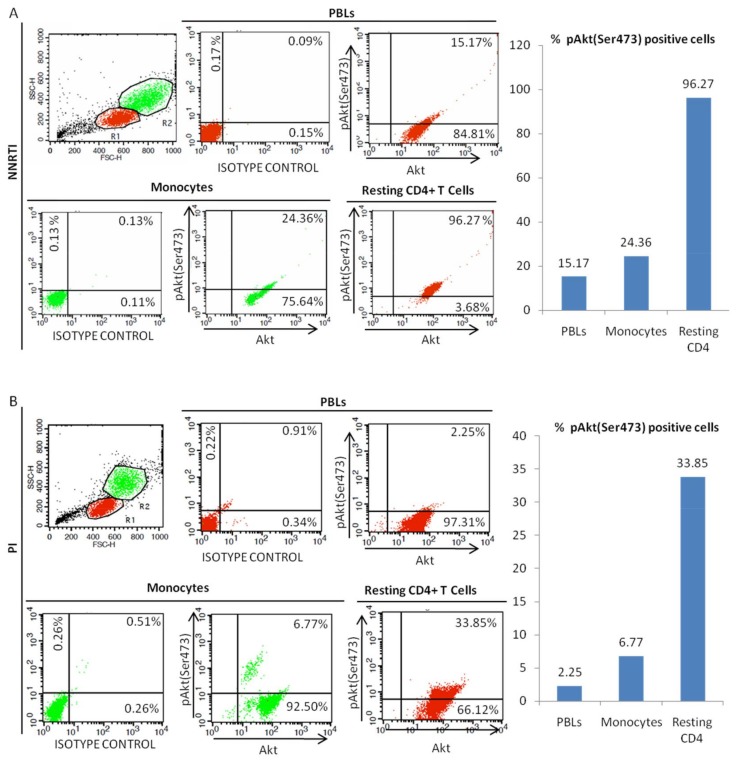
Levels of Akt activation in peripheral blood lymphocytes (PBLs), monocytes, and resting CD4+ T cells from aviremic HIV-1 patients under cART. A,B. Akt activation was lower in PBLs and monocytes than in resting CD4+ T cells isolated from (**A**) a NNRTI-treated patient and (**B**) a PI-treated patient as measured by flow cytometric analysis. Results are representative of 23 independent experiments.

**Figure 5:**
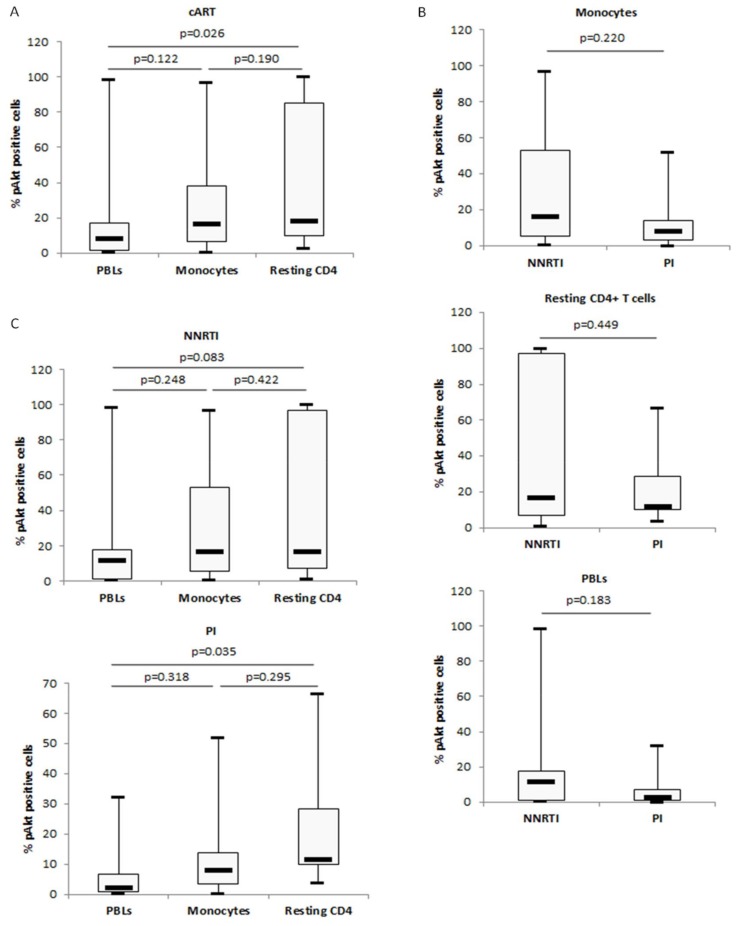
Impact of cART, PI-, and NNRTI-treatment on Akt activation in monocytes, resting CD4+ T cells, and PBLs isolated from aviremic HIV-1-infected patients. (**A**) Percentage of P-Akt positive cells in PBLs, monocytes, and autologous resting CD4+ T cells isolated from cART-treated patients (*n* = 23) as measured by flow cytometric analysis. The histogram shows median and quartiles. (**B**) Percentage of P-Akt positive cells depends on the cell type tested and on the cART treatment used (PI versus NNRTI) as measured by flow cytometric analysis. The histogram shows median and quartiles (*n* = 23). (**C**) Respective percentages of P-Akt positive PBLs, monocytes, and resting CD4+ T cells from aviremic patients under PI-cART and NNRTI-cART as measured by flow cytometric analysis. The histogram shows median and quartiles (*n* = 23).

In HIV-1-positive patients under cART, Akt activation was lower in monocytes compared to autologous resting CD4+ T cells with the lowest activation in the bulk of PBLs (Figure 4 and Figure 5A) (Table 1). The lowest Akt activation was observed in aviremic patients under PI-cART rather than under NNRTI-cART, and primarily in monocytes and PBLs rather than in autologous resting CD4+ T cells (Figure 5B) (Table 1). PI-cART was more efficient for decreasing Akt activation than NNRTI-cART irrespective of the cell type tested (Figure 5C) (Table 1). Altogether, our results indicate that PI-cART treatment is the most potent to decrease Akt activation especially in monocytes isolated from aviremic patients.

### 3.5. Limited Amounts of HIV-1 Proviral DNA in Monocytes from Aviremic Patients under Cart

**Figure 6:**
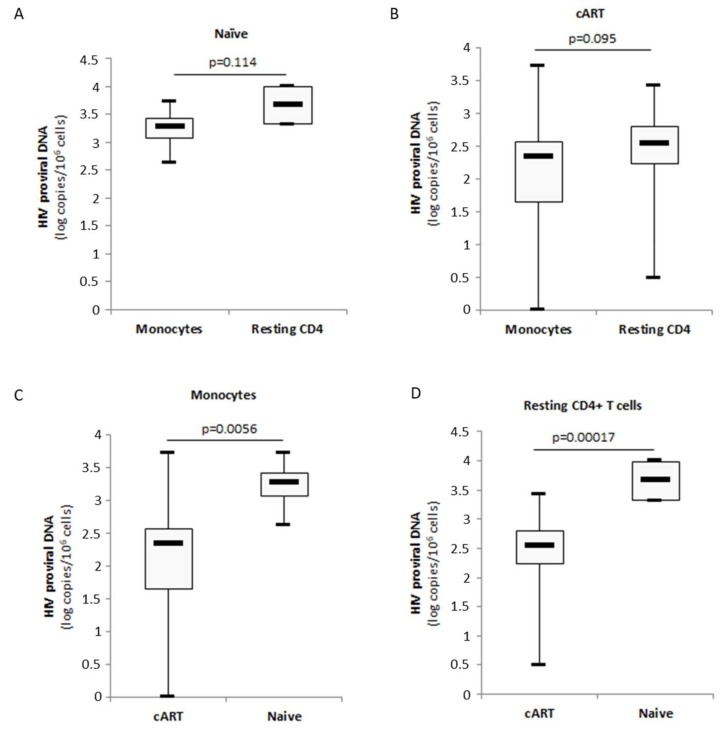
Lower amounts of proviral DNA in monocytes and resting CD4+ T cells from cART-treated patients compared to cART-naïve patients. HIV-1 proviral DNA was measured in monocytes and autologous resting CD4+ T cells isolated from cART-treated patients and cART-naïve patients using a Biocentric Generic HIV DNA Cell kit. (**A**) Amounts of HIV-1 proviral DNA in monocytes compared to autologous resting CD4+ T cells isolated from cART-naïve patients (*n* = 4). The histogram shows median and quartiles. (**B**) Amounts of HIV-1 proviral DNA in monocytes compared to autologous resting CD4+ T cells isolated from cART-treated patients (*n* = 31). The histogram shows median and quartiles. (**C**) Lower amounts of HIV-1 proviral DNA in monocytes from cART-treated patients compared to cART-naïve patients (*n* = 31). The histogram shows median and quartiles. (**D**) Lower amounts of HIV-1 proviral DNA in resting CD4+ T cells from cART-treated patients compared to cART-naïve patients. The histogram shows median and quartiles (*n* = 31).

**Figure 7:**
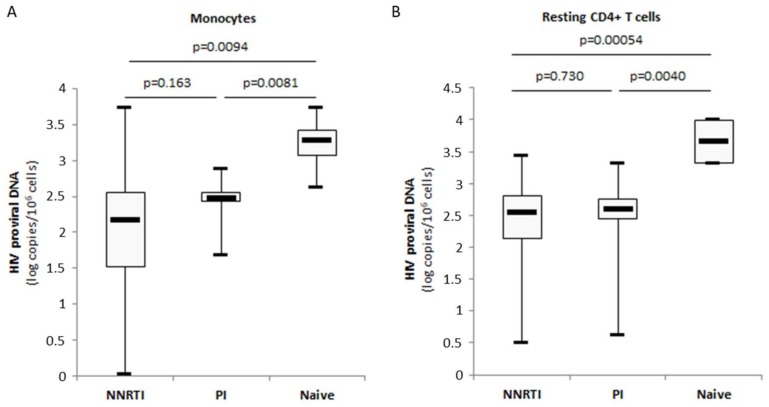
cART reduces the amount of HIV proviral DNA in monocytes and resting CD4+ T cells from aviremic patients irrespective of the PI or NNRTI treatment. (**A**) Lower amounts of HIV-1 proviral DNA in monocytes from patients under PI-cART or NNRTI-cART compared to cART-naïve patients. The histogram shows median and quartiles (*n* = 31). (**B**) Lower amounts of HIV-1 proviral DNA in resting CD4+ T cells from patients under PI-cART or NNRTI-cART compared to cART-naïve patients. The histogram shows median and quartiles (*n* = 34).

Besides resting CD4+ T cells, latently infected monocytes/macrophages that harbour integrated replication-competent viral DNA represent a primary long-lived source of persistent HIV-1 in patients under cART [27,28,29]. Therefore, we decided to analyze the respective sizes of the HIV-1 reservoirs in monocytes and resting CD4+ T cells from patients naïve of treatment and the impact of the cART regimen on the viral reservoir in monocytes and autologous resting CD4+ T cells by quantifying total proviral HIV-1 DNA. The amount of HIV-1 proviral DNA was lower in monocytes compared to autologous resting CD4+ T cells isolated from cART naïve patients (3.2 log versus 3.7 log, *p* = 0.114) (Figure 6A). A similar lower amount of HIV-1 proviral DNA was measured in monocytes compared to resting CD4+ T cells from cART-treated patients (2.2 log versus 2.4 log, *p* = 0.095) (Figure 6B). Under cART, the amount of HIV-1 proviral DNA present in monocytes and resting CD4+ T cells was decreased significantly compared to cART-naïve patients (*p* = 0.0056 and *p* = 0.00017, respectively) (Figure 6C,D). In PI-treated patients but also in NNRTI-treated patients, significantly lower amounts of proviral DNA were measured in monocytes (*p* = 0.0081 and *p* = 0.0094, respectively) and resting CD4+ T cells (*p* = 0.0040 and *p* = 0.00054, respectively) compared to cART-naïve patients (Figure 7A,B). We did not find significant difference for the amount of proviral DNA present in monocytes and resting CD4+ T cells between PI-cART- and NNRTI-cART-treated patients (Figure 7A,B) (Table 1).

## 4. Discussion

HIV-1 Nef, a pleiotropic early viral protein, has been demonstrated to activate several key signaling molecules, including Akt, MAPK, and ERK [45,46]. In the present study, we have demonstrated that Nef upregulates Akt expression and induces Akt phosphorylation in MDMs in vitro, which was blocked by the HIV PI lopinavir/ritonavir but not by the NNRTI nevirapine. Both increased Akt activation and enhanced HIV-1 reactivation were observed in U1 monocytoid cells stimulated with TNF. The HIV-1 reactivation in U1 cells stimulated by TNF was inhibited by the Akt inhibitor VIII and the PI lopinavir/ritonavir, but not by the NNRTI nevirapine. Most importantly, our results indicate that the PIs, but not the NNRTIs, decrease Akt hyperactivation in vitro and in vivo. Both PI-cART and NNRTI-cART limit the size of the HIV-1 reservoir in latently infected monocytes from aviremic chronically infected patients.

Exogenous HIV-1 Nef is internalized by MDMs as reported previously for CD4+T cells [20,47]. The mode of internalization and secretion of Nef is still a conundrum. Some studies suggest the involvement of exosomes in intercellular transfer of Nef from infected cells to the bystander cells [48,49]. On the other hand, a recently published study suggests the cell-to-cell contact rather than exosomes-mediated intercellular transfer of Nef [50]. In vivo, exogenous Nef and TNF secreted by infected cells as soluble proteins and/or as exosomes can activate uninfected CD4+ T cells and MDMs present in the vicinity and make them susceptible to infection [31,39,51]. Following Nef treatment, a 2-fold increase in NF-kB-mediated LTR activation in U937 cells transfected with LTR-Luc and also a 2-fold increase of p24 levels in supernatants of chronically HIV-infected U1 cells have been previously described [39,52,53]. In U1 cells treated with TNF, both NF-kB activation and HIV-1 reactivation from latency are observed [40,53]. Thus, both Nef and TNF could activate monocytes/macrophages and thereby expand the viral reservoir in infected individuals.

Latency represents the major obstacle in the complete cure of HIV-1 [25,54,55,56]. Several agents targeting various signaling cascades are known to reactivate latent HIV-1 and are termed latency-reversing agents (LRAs) [57,58]. Among LRAs, activators of the PI3K/Akt pathway could be used to reactivate the HIV-1 latent reservoir in both lymphoid and myeloid cells. For instance, disulfiram is known to deplete phosphatase and tensin homolog (PTEN) protein levels in U1 cells and in resting CD4+ T cells from HIV-negative donors, resulting in hyperphosphorylation of Akt and subsequently activation of HIV-1 expression [59]. In another instance, 57,704, an agonist of PI3K, reactivates HIV-1 in several latent cell lines [59]. LRAs expose latently infected cells to the host immune system and to cART, namely the “shock and kill” strategy. This strategy faces multiple barriers which prevent the complete eradication of replication-competent viruses and must therefore be optimized. Recently, a “block and lock” strategy has been hypothesized which focuses on suppressing residual levels of HIV-1 transcription to lock the virus in a deep latency state to prevent viral reactivation [25]. In view of the “block and lock” strategy, Akt inhibitors could be suggested as potential therapeutic molecules against HIV-1 especially in infected monocytes/macrophages [60]. The role of PIs, but not RTIs, in inhibiting Akt activation has been shown in several cell types [15,18,60,61,62]. We observed that PIs could block Nef-mediated Akt activation in MDMs in a dose-dependent manner (Figure 2). Furthermore, PIs inhibit the viral reactivation from chronically infected monocytoid U1 cells stimulated with TNF similar to Akt inhibitor VIII (Figure 3). Thus, the inhibition of viral reactivation by PIs could participate in the “block and lock” strategy and thereby curtail the HIV-1 reservoir especially in myeloid cells.

Although the use of cART has been extensively studied in regard to residual viremia, only little is known on their impact on cellular reservoirs of HIV-1 especially in monocytes isolated from patients. We determined the levels of Akt activation and HIV-1 proviral DNA in monocytes and autologous resting CD4+ T cells from cART-treated patients. In cART-naïve patients, we observed that the amount of HIV-1 proviral DNA was 0.5 log smaller in monocytes than in resting CD4+ T cells (Figure 6). In agreement with our results, the number of productively infected monocytes is nearly 10-fold lower than the number of productively infected CD4+ T cells in the blood of Simian Immunodefiency Virus-infected macaques [63]. In agreement with decreased Akt activation in resting CD4+ T cells from PI-treated patients [21], we observed low levels of Akt activation in monocytes isolated from PI-treated patients. By using the proviral HIV DNA assay that allows for evaluating in a quantitative manner the amount of intracellular provirus, we observed that cART was efficient for limiting the size of the HIV-1 reservoir in monocytes and resting CD4+ T cells compared to cART-naïve patients (Figure 6). To diminish the size of the viral reservoir, several studies have shown that an early treatment during HIV-1 primo-infection could be beneficial [33,34], and on-demand pre-exposure prophylaxis has been shown to be highly efficient to prevent HIV transmission [64]. In addition, PIs have been reported to block the cell-to-cell spread of HIV-1 [65]. Since myeloid cells are a main target of macrophage-tropic HIV-1 strains following HIV exposure, future studies will have to assess the effect of on-demand pre-exposure prophylaxis not only on CD4+ T cells but also on the monocyte/macrophage population subset.

In addition to its role in HIV-1 reactivation and latency, Akt activation is critical for cell survival [1]. Therefore, the blockade of Akt activation by PIs in latently infected resting monocytes/macrophages could trigger an accelerated cell death and thereby favour the clearance of the HIV reservoir in myeloid cells [19]. In fact, HIV- and SIV-infected macrophages are not efficiently killed by CD8+ T cells like infected CD4+ T cells are [66,67]. Also, resident tissue macrophages remain in tissues long term, are relatively resistant to the cytopathic effects of HIV infection compared to CD4+ T cells, and may serve as stable viral reservoirs [68]. In addition, the relative potency of PIs in chronically infected monocytes/macrophages could be a limiting factor for the clearance of the virus in myeloid cells [69,70,71]. Therefore, besides their antiviral activity, PIs rather than NNRTIs could, by targeting Akt, trigger apoptosis in HIV-infected monocytes/macrophages and neighbouring CD4+ T cells [72,73] and thereby participate in the clearance of both myeloid and lymphoid infected cells.

## 5. Conclusions

Our study shows that exposure of MDMs to HIV-1 Nef leads to enhanced Akt expression and activation. Importantly, we observed that the immunomodulatory effects of PIs, by blocking Akt activation especially triggered by Nef and TNF, limit HIV-1 recovery from latently infected monocytoid U1 cells in vitro. Finally, in aviremic chronically HIV-1-infected patients, our study indicates that PI-based cART decreases Akt activation and limits the size of the monocyte reservoir. Altogether, our results support the need to further assess the therapeutic use of HIV-1 protease inhibitors in view of their impact on persistence of myeloid HIV-1 reservoirs and might provide and/or accompany new therapeutic strategies for a remission.

## Figures and Tables

**Table 1:** Biological and virological characteristics of HIV-1-positive patients under PI-cART and NNRTI-cART.

	PI	NNRTI	
Biological Characteristics	*n* = 8	*n* = 23	*p*-Value
Age (years), mean (±s.d.)	42.2 (10.5)	49 (7.3)	0.136
CD4+ T cell counts at nadir, absolute (cells per µL), median (IQR)	255.5 (185.7–368.8)	288.8 (94.6–362.6)	0.734
CD4+ T cell counts at initiation of treatment, absolute (cells per µL), median (IQR)	361.8 (300.5–530.5)	332.3 (128.5–425.4)	0.407
CD4+ T cell counts at the last point, absolute (cells per µL), median (IQR)	614 (552.5–664.1)	761.6 (492–934.4)	0.288
pVL at zenith (log copies per mL), median (IQR)	5.10 (4.40–5.26)	4.98 (4.26–5.53)	0.982
Previous treatment failure, median (IQR)	1.5 (0–6)	1 (0–2.5)	0.694
Time with therapy (years), mean ± s.d.	11.6 (8)	9 (4.7)	0.498
Time with undetectable pVL (years), mean ± s.d.	5.8 (3.2)	6.5 (3.8)	0.804
**Akt Activation Assay**			
**Resting CD4+ T Cells**			
% of pAkt(Ser473) positive cells, mean ± s.d.	22.8 (23.8)	52.2 (43.5)	0.449
**Monocytes**			
% of pAkt(Ser473) positive cells, mean ± s.d.	13.5 (18.0)	32.1 (33.0)	0.220
*p*-value, monocytes versus resting CD4+ T cells	0.295	0.442	
**Proviral HIV DNA quantification Assay**			
**Resting CD4+ T Cells**			
Proviral HIV-1 DNA (log copies per 10^6^ cells),median (IQR)	2.58 (2.45–2.81)	2.53 (2.04–2.79)	0.730
**Monocytes**			
Proviral HIV-1 DNA (log copies per 10^6^ cells), median (IQR)	2.47 (2.43–2.55)	2.12 (1.43–2.53)	0.163
*p*-value, monocytes vs resting CD4+ T cells	0.462	0.129	

IQR = interquartile range.
